# Measuring HIV Acquisitions Among Partners of Key Populations: Estimates From HIV Transmission Dynamic Models

**DOI:** 10.1097/QAI.0000000000003334

**Published:** 2024-01-04

**Authors:** Romain Silhol, Rebecca L. Anderson, Oliver Stevens, James Stannah, Ross D. Booton, Stefan Baral, Dobromir Dimitrov, Kate M. Mitchell, Deborah Donnell, Anna Bershteyn, Tim Brown, Sherrie L. Kelly, Hae-Young Kim, Leigh F. Johnson, Mathieu Maheu-Giroux, Rowan Martin-Hughes, Sharmistha Mishra, Wiwat Peerapatanapokin, Jack Stone, John Stover, Yu Teng, Peter Vickerman, Sonia Arias Garcia, Eline Korenromp, Jeffrey W. Imai-Eaton, Marie-Claude Boily

**Affiliations:** a MRC Centre for Global Infectious Disease Analysis, School of Public Health, Imperial College London, London, United Kingdom;; b HIV Prevention Trials Network Modelling Centre, Imperial College London, London, United Kingdom;; c Department of Epidemiology and Biostatistics, School of Population and Global Health, Faculty of Medicine and Health Sciences, McGill University, Montréal, Quebec, Canada;; d Department of Epidemiology, Johns Hopkins Bloomberg School of Public Health, Baltimore, MD;; e Vaccine and Infectious Disease Division, Fred Hutchinson Cancer Center, Seattle, WA;; f Department of Nursing and Community Health, Glasgow Caledonian University London, London, United Kindom;; g Department of Population Health, New York University Grossman School of Medicine, New York, New York;; h Research Program, East-West Center, Honolulu, HI;; i Burnet Institute, Melbourne, Victoria, Australia;; j Centre for Infectious Disease Epidemiology and Research, School of Public Health, University of Cape Town, Cape Town, South Africa;; k Department of Medicine, University of Toronto, Toronto, Ontario, Canada;; l Population Health Sciences, Bristol Medical School, University of Bristol, Bristol, United Kingdom;; m Avenir Health, Glastonbury, CT;; n Data for Impact Division, UNAIDS, Geneva, Switzerland; and; o Center for Communicable Disease Dynamics, Department of Epidemiology, Harvard T. H. Chan School of Public Health, Boston, MA.

**Keywords:** key populations, female sex workers, clients of female sex workers, men who have sex with men, people who inject drugs, transgender women, HIV incidence

## Abstract

**Background:**

Key populations (KPs), including female sex workers (FSWs), gay men and other men who have sex with men (MSM), people who inject drugs (PWID), and transgender women (TGW) experience disproportionate risks of HIV acquisition. The UNAIDS Global AIDS 2022 Update reported that one-quarter of all new HIV infections occurred among their non-KP sexual partners. However, this fraction relied on heuristics regarding the ratio of new infections that KPs transmitted to their non-KP partners to the new infections acquired among KPs (herein referred to as “*infection ratios”*). We recalculated these ratios using dynamic transmission models.

**Setting:**

One hundred seventy-eight settings (106 countries).

**Methods:**

*Infection ratios* for FSW, MSM, PWID, TGW, and clients of FSW were estimated from 12 models for 2020.

**Results:**

Median model estimates of *infection ratios* were 0.7 (interquartile range: 0.5–1.0; n = 172 estimates) and 1.2 (0.8–1.8; n = 127) for acquisitions from FSW clients and transmissions from FSW to all their non-KP partners, respectively, which were comparable with the previous UNAIDS assumptions (0.2–1.5 across regions). Model estimates for female partners of MSM were 0.5 (0.2–0.8; n = 20) and 0.3 (0.2–0.4; n = 10) for partners of PWID across settings in Eastern and Southern Africa, lower than the corresponding UNAIDS assumptions (0.9 and 0.8, respectively). The few available model estimates for TGW were higher [5.1 (1.2–7.0; n = 8)] than the UNAIDS assumptions (0.1–0.3). Model estimates for non-FSW partners of FSW clients in Western and Central Africa were high (1.7; 1.0–2.3; n = 29).

**Conclusions:**

Ratios of new infections among non-KP partners relative to KP were high, confirming the importance of better addressing prevention and treatment needs among KP as central to reducing overall HIV incidence.

## Abstract 2

Supplemental Digital Content is Available in the Text.

## INTRODUCTION

Key populations (KPs) have been disproportionately affected by increased risk of HIV acquisition and transmission since the beginning of the HIV pandemic. KPs include female sex workers (FSWs), gay men and other men who have sex with men (MSM), people who inject drugs (PWID), and transgender women (TGW). Moreover, their unmet HIV prevention and treatment needs are intertwined with overall HIV transmission dynamics, and addressing these needs is central to an effective HIV response.^1–3^ To achieve this, understanding HIV transmission risks between KPs and their sexual partners that do not belong to these groups (ie, non-KP) is important.

Each year, the Joint United Nations Programme on HIV/AIDS (UNAIDS) reports estimates for the proportion of annual new HIV infections (NIs) acquired by KPs and their non-KP partners, by global region (see Table 1, Supplemental Digital Content, http://links.lww.com/QAI/C158).^4^ These estimates, based on collated national-level statistical or transmission dynamic trend estimates or country-reported new diagnoses by mode of transmission, are highly cited to advocate for appropriate prevention and treatment access among population groups most affected by HIV.^5^ NIs among non-KP partners of KPs (eg, clients of FSW and female partners of MSM) were estimated to represent a quarter of all NIs in the UNAIDS 2022 Global AIDS Update,^4^ when calculated assuming fixed ratios (referred to here as “*infection ratios*”) reflecting simplistic assumptions regarding HIV transmissions from KPs to their non-KP partners. These KP- and region-specific *infection ratios* are defined as the number of NIs in non-KP partners of each KP divided by the number of NIs in each KP. The ratios were estimated heuristically by the UNAIDS for each global region in 2016 from a nonsystematic review of the expected numbers of non-KP partners of each KP population (eg, average annual number of cisgender female sex partners of MSM or the annual number of non-PWID sexual partners of PWID), with further adjustments being made to only account for transmission rates during newly acquired HIV infection in different partnership types from Patel et al.^6^ Empirically determining these ratios and the number of NIs occurring among non-KP partners of KPs is challenging because these populations are difficult to reach through population-based surveys and empirically measuring transmission events is usually not feasible.

The *infection ratio* approach used by the UNAIDS in 2022 to estimate NIs among non-KP partners of KP reflects the magnitude of the additional indirect benefits of addressing KPs treatment needs. It is simple and useful when data are limited but has substantial shortcomings: it does not account for all people living with HIV (only those having recently acquired the infection are assumed to transmit HIV) and does not vary by epidemic situation (ie, does not include a time dimension). Furthermore, the ratio could not be calculated systematically for all KPs in all regions due to data gaps and does not consider acquisitions among partners of KPs due to sex with other non-KPs (eg, nonclient male partners of FSW). Importantly, the UNAIDS approach for 2022 has not previously been formally compared with alternative estimation methods, notably dynamic HIV transmission modelling that relies on comprehensive epidemiological data reflecting KP-specific epidemiology, risk behaviors, and prevention/treatment coverage over time (and provides infection distributions over time).

We used results from 12 different dynamic models of HIV transmission across different settings (including the *Goals*^7^ or *AEM*^8^ models, on which most updated UNAIDS 2023 HIV estimates rely^5^) to compare model-based estimates of *infection ratios* (ie, transmissions from KPs to their non-KP partners divided by the NIs acquired by the referent KP) with the ratios assumed by the UNAIDS in corresponding regions. The 3 main questions addressed by our study were as follows: (1) How do model-based estimates of the *infection ratios* compare with the assumptions made for the UNAIDS 2022 Global AIDS Update?^4^ (2) Do these ratios vary by region and time? and (3) To what extent are there differences between estimated ratios due to different models or methods?

Our analysis also considered infections among FSW clients and their non-KP partners (counted by the UNAIDS in the “remaining population”), given emerging evidence that FSW clients account for substantial fractions of NIs in sub-Saharan Africa (SSA), for example around 40% in Côte d’Ivoire and South Africa.^9,10^

As a result of our analysis presented here (and of a study of empirically derived or model-derived estimates of fractions of new HIV infections occurring among KPs^11^), the UNAIDS has refined their methodology underpinning their annual estimates by replacing the time-constant *infection ratios* by time-varying non-KP partner infection estimates directly from transmission dynamic models (a subset of the same models we analyzed here).^5^

## METHODS

### Mathematical Models and KPs Included

**FIGURE 1.**
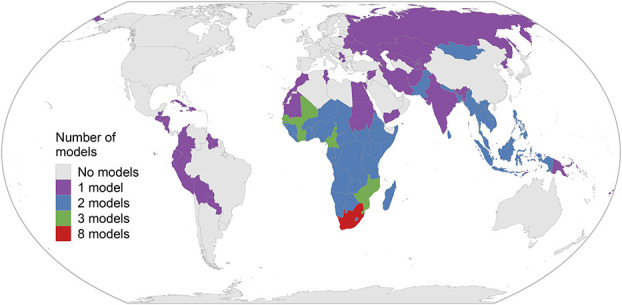
World map of countries for which model-based estimates of the *infection ratios* were used for our analysis. Colors represent the number of transmission-dynamic models that had simulated the HIV epidemic in the country and included at least 1 KP and their sexual partners and were available for this analysis.

We calculated estimates of the *infection ratios* from 178 applications (“settings”) of 12 existing transmission-dynamic models, which were provided by 9 modelling groups, capturing 106 different countries (Fig. 1, Table 1). Most models were calibrated to empirical country-specific HIV epidemiological and intervention data, including KP survey data.

**TABLE 1.** Characteristics of the Dynamic Models of HIV Transmission and Estimates of the *Infection Ratios* Used for This Analysis

Model Name (Model Type)	No. of Settings* Modeled	Regions Modeled	KPs and High-Risk Populations Modeled (No. of Settings With Available Estimates of the Numbers of Acquisitions by Their Non-KP Partners (A) or Transmissions to Their Non-KP Partners (T)† and Year Estimates Available	Method Used to Quantify Transmissions From MSM or FSW Clients to Their Non-KP Partners (Direct Transmissions vs 1-yr Counterfactual-Based Estimates)	Comments
Goals 2022^7^ (deterministic compartmental)	106	AP	FSW (A: 100/106; T: 99/106)	Direct transmissions	Applied KP size estimates reported by countries to UNAIDS within 2017–2021.
		CAR	MSM (A: 0/106; T: 95/106)		MSM are assumed to form stable and nonregular partnerships with other men, and only stable partnerships with females.
		EECA	PWID (A: 0/106; T: 69/106)		People sharing multiple risk factors (eg, those injecting drugs also having multiple partners) are assigned to the group with the highest risk of HIV acquisition.
		ESA	FSW clients (A: 0/106; T: 100/106)		
		LA	Available for 2022 (and assumed for 2020) only		
		MENA			
		WCA			
		WCE			
*Goals Global Fund (GF)* 2022^12^ (deterministic compartmental)	44	ESA	FSW (A: 44/44; T: 0/44)	No estimates of the number of transmissions available	Considered KP size estimates reported by countries to the UNAIDS within 2015–2021: the median sizes of the FSW population across the Goals GF models were ∼1.3-fold lower and 1.1-fold higher in ESA and WCA compared with Goals 2022, respectively. Median sizes of the MSM population were 2-fold higher and 2-fold lower in ESA and WCA, respectively. Median sizes of the PWID population were 1.9-fold higher and 4-fold lower in ESA and WCA, respectively.
		MENA‡	MSM (A: 0/44; T: 0/44)		
		WCA	PWID (A: 0/44; T: 0/44)		
			Available for 2020 only		
*Goals HPTN*^13^ (deterministic compartmental)	1	ESA	FSW (A: 1/1; T: 1/1)	Direct transmissions	Model for South Africa only, outputted for the HIV prevention trials network (HPTN).
			MSM (A: 0/1; T: 1/1)		
			PWID§ (A: 0/1; T: 0/1)		
			FSW clients (A: 0/1; T: 1/1)		
			Available for 2010 and 2020		
AEM^8^ (deterministic compartmental)	13‖	AP	FSW (A: 13/13; T: 13/13)	Direct transmissions	Specific KP sizes were set to 0 in models where KP-specific data were deemed insufficient.
			MSM (A: 0/13; T: 13/13)		FSW and TGW selling sex for money were combined. The fraction of infections acquired by FSW included acquisitions during drug injecting. Acquisitions among and transmissions from MSM included acquisitions/transmissions from male sex workers.
			PWID§ (A: 0/13; T: 12/13)		
			TGW§ (A: 0/13; T: 8/13)		
			Available for 2010 and 2020		
Optima^14^ (deterministic compartmental)	5	ESA	FSW (A: 5/5; T: 5/5)	Counterfactual-based	
			MSM (A: 0/5; T: 2/5)		
			FSW clients (A: 0/5; T: 5/5)		
			Available for 2010 and 2020		
Thembisa^15^ (deterministic compartmental)	1	ESA	FSW (A: 1/1; T: 1/1)	Counterfactual-based	Clients of FSW include former clients.
			MSM (A: 0/1; T: 1/1)		MSM are assumed to have 30% of their sexual contacts with women.
			FSW clients (A: 0/1; T: 1/1)		
			Available for 2010 and 2020		
EMOD^16^ (stochastic individual-based)	1	ESA	FSW (A: 1/1; T: 1/1)	Counterfactual-based	
			Available for 2010 and 2020		
Stone et al^10^ (deterministic compartmental)	1	ESA	FSW (A: 1/1; T: 1/1)	Counterfactual-based	MSM population is stratified into 2 age groups.
			MSM (A: 0/1; T: 1/1)		
			FSW clients (A: 0/1; T: 1/1)		
			Available for 2010 and 2020		
Mishra^17^ (deterministic compartmental)	1	ESA	FSW (A: 1/1; T: 1/1)	Counterfactual-based	The modeled population combines South Africa, Eswatini, and Lesotho.
			Available for 2010 and 2020		Differentiates current FSW and former FSW to better inform sex-work turnover.
Maheu-Giroux^9^ (deterministic compartmental)	1	WCA	FSW (A: 1/1; T: 1/1)	Counterfactual-based	Differentiates MSM ever having female partners and MSM only having male partners.
			MSM (A: 0/1; T: 1/1)		
			Available for 2010 and 2020		
Silhol Yaoundé^18^ (deterministic compartmental)	1	WCA	FSW (A: 1/1; T: 1/1)	Counterfactual-based	
			MSM (A: 0/1; T: 1/1)		
			FSW clients (A: 0/1; T: 1/1)		
			Available for 2010 and 2020		
Silhol–ATLAS^19^ (deterministic compartmental)	3	WCA	FSW (A: 3/3; T: 3/3)	Both direct transmissions and counterfactual-based estimates available	Differentiates MSM ever having female partners and MSM only having male partners.
			MSM (A: 3/3; T: 3/3)		
			FSW clients (A: 0/3; T: 3/3)		
			Available for 2010 and 2020		

* All settings are full countries except in the model by Mishra et al, which combines South Africa, Eswatini, and Lesotho, and the Silhol Yaoundé model, which only represents Cameroon’s capital city.

† The difference between approach A (A) and approach T (T) is the inclusion of client infections from other non-KP partners in (A) in addition to those from FSW, whereas (T) includes FSW transmissions to nonclient partners and those of clients. See Methods section and supplement.

‡ One country, Djibouti, located in SSA.

§ Infection ratios estimates for PWID from AEM include transmissions from FSW-PWID to their non-KP partners, whereas estimated for TGW include transmissions from FSW-TGW to their non-KP partners.

‖ AEM estimates were used for 13 countries: Bangladesh, Cambodia, Indonesia, Lao PDR, Malaysia, Mongolia, Myanmar, Nepal, Pakistan, Philippines, Sri Lanka, Thailand, and Vietnam.

CAR, Caribbean; EECA, Eastern Europe and Central Asia; LA, Latin America; MENA, Middle East and North Africa; WCE, Western and Central Europe.

Table 1 summarizes key characteristics of each model, including the settings and risk populations represented. Most estimates were calculated from the deterministic compartmental *Goals* model^7^ (151/178), which considers FSW, MSM, PWID, clients of FSW, and other non-KP groups. Three sets of *Goals* estimates were available and used for our analysis across 8 regions (presented as different models). First, we used outputs from the version of *Goals* used for the 2022 Global AIDS Update (“Goals 2022” estimates) covering 106 countries. We also used *Goals* outputs for 44 SSA countries produced for a specific planning collaboration with the Global Fund (GF) (“*Goals GF*” estimates).^12^ The main difference between the *Goals 2022* and *Goals GF* estimates for SSA are the assumed size of KP used in the models (Table 1). Finally, *Goals* estimates were computed for South Africa in 2019 for an independent analysis in collaboration with the HIV Prevention Trials Network Modelling Centre (“Goals HPTN” estimates).^13^

The AIDS Epidemic Model (*AEM*),^8^ which represents FSW, MSM, PWID, TGW, clients of FSW, and other non-KP groups, provided country-vetted estimates for 13 settings in Asia from the 2023 UNAIDS-supported HIV estimation round. *Optima*,^14^ which considers FSW, MSM, PWID, clients of FSW, and non-KP groups, provided estimates for 5 settings in Eastern and Southern Africa (ESA), using models parametrized and calibrated in 2019. *Thembisa*^15^ and Stone et al^10^ provided estimates for FSW, MSM, clients of FSW, and other non-KP groups in South Africa. *EMOD*^16^ represented FSW, clients of FSW, and non-KPs in South Africa. The model in the study by Mishra et al^17^ provided outputs for FSW, their clients, and other non-KPs in South Africa, Eswatini, and Lesotho combined.

For West and Central African (WCA) settings, the model from Maheu-Giroux et al provided estimates for FSW, MSM, clients of FSW, and other non-KP groups in Côte d’Ivoire.^9,20^ Two models from Silhol et al considered FSW, MSM, clients of FSW, and other non-KP groups, in Yaoundé (capital city of Cameroon)^18^ and 3 West African countries (“ATLAS”: Côte d’Ivoire, Mali, and Senegal).^19^

### Overview of Settings and KPs Modeled

We calculated *infection ratios* for the year 2020 in all models, except for *Goals 2022*, which was derived for 2022. Of the 178 modeled settings, most (102) were in SSA, including 50 in ESA and 52 in WCA (Fig. 1 and Table 2). Only 1 estimate for the Western and Central Europe region was available (from *Goals*), and none for North America. Table 2, Supplemental Digital Content, http://links.lww.com/QAI/C158 reports the number of settings for which model-based estimates of the *infection ratio* were calculated, stratified by region/model combinations. Most models other than Goals, AEM, and Optima were for South Africa.

**TABLE 2.** Median and Interquartile Range (25^th^ and 75^th^ Percentiles of Estimates, and n = No. of Estimates for the Setting) of Model-Based Estimates of the *Infection Ratio* for Clients and Non-KP Partners of KPs for the Year 2020, Alongside Assumptions Made by the UNAIDS About Year 2021 in the Global AIDS Update 2022

Region*	Clients and Partners of FSW (Acquisitions by Clients, Approach A†)	Clients and Partners of FSW (Transmissions From FSW, Approach T†)	Female Partners of MSM (Transmissions From MSM)	Non-KP Partners of PWID (Transmissions From PWID)	Non-KP Partners of TGW (Transmissions From TGW)	Non-KP Partners of FSW Clients (Transmissions From Clients of FSW)
All	0.7 (0.5–1.0; n = 172)	1.2 (0.8–1.8; n = 127)	0.4 (0.2–0.6; n = 118)	0.3 (0.2–0.6; n = 81)	5.1 (1.2–7.0; n = 8)	1.1 (0.6–1.9; n = 112)
Asia and Pacific						
Model estimates	0.8 (0.5–1.6; n = 33)	1.4 (1.1–2.1; n = 32)	0.2 (0.1–0.3; n = 35)	0.2 (0.2–0.5; n = 30)	5.1 (1.2–7.0; n = 8)	1.2 (1–2.2.0; n = 21)
UNAIDS assumption	0.25‡		0.15	0.25	0.1	NA
Caribbean						
Model estimates	0.5 (0.4–0.6; n = 7)	0.8 (0.7–1.1; n = 7)	0.5 (0.5–0.6; n = 7)	0.2 (0.2–0.3; n = 3)	n = 0	0.6 (0.4–1.9; n = 6)
UNAIDS assumption	0.70‡		0.5	0.8	0.1	NA
Eastern Europe and Central Asia						
Model estimates	0.6 (0.5–0.9; n = 10)	0.8 (0.6–1.5; n = 10)	0.3 (0.2–0.6; n = 12)	0.7 (0.4–1.1; n = 10)	n = 0	0.5 (0.4–0.6; n = 12)
UNAIDS assumption	0.2‡		0.05	0.35	0.1	NA
Eastern and Southern Africa						
Model estimates	0.8 (0.6–1.3; n = 49)	1.4 (0.7–1.9; n = 29)	0.5 (0.2–0.8; n = 20)	0.3 (0.2–0.4; n = 10)	n = 0	1.1 (0.7–1.7; n = 27)
UNAIDS assumption	1.5‡		0.9	0.8	0.1	NA
Latin America						
Model estimates	0.6 (0.5–0.8; n = 10)	1.3 (1–1.5; n = 10)	0.4 (0.2–0.5; n = 10)	0.4 (0.2–0.4; n = 5)	n = 0	1.0 (0.8–1.2; n = 8)
UNAIDS assumption	1.5‡		0.3	0.9	0.3	NA
Middle East and North Africa						
Model estimates	0.8 (0.7–0.9; n = 11)	1.0 (0.9–1.5; n = 9)	0.1 (0.1–0.3; n = 7)	0.3 (0.2–0.5; n = 7)	n = 0	1.0 (0.8–1.2; n = 9)
UNAIDS assumption	0.3‡		0.1	0.3	0.1	NA
Western and Central Africa						
Model estimates	0.6 (0.5–0.8; n = 51)	1.2 (0.9–1.5; n = 29)	0.5 (0.4–1; n = 26)	0.3 (0.2–0.5; n = 15)	n = 0	1.7 (1.0–2.3; n = 29)
UNAIDS assumption	0.75‡		0.5	0.8	0.1	NA
Western and Central Europe						
Model estimates	0 (n = 1)	1.0 (n = 1)	1.5 (n = 1)	0.6 (n = 1)	n = 0	n = 0
UNAIDS assumption	0.35‡		0.05	0.5	0.1	NA

* No model-based estimate was available for the North American region.

† The difference in the 2 approaches is the inclusion of FSW client's acquisitions from other non-KP partners in approach A in addition to those from FSW, whereas approach T includes FSW transmissions to nonclient partners and those of clients.

‡ The UNAIDS assumption for all SW, however, almost exclusively informed by data on FSW.

All models explicitly represented FSW (12/12) and most represented MSM (10/12). Only 4 of 12 included PWID, and information about their transmissions to non-KP partners was often not available (Table 1). Estimates for TGW were only available from *AEM* (for 8/13 Asian and Pacific countries) and included transmissions from TGW sex workers (SW). All models explicitly represented clients of FSW (12/12), and *infection ratios* estimates for their non-KP partners were available from 7 models (Table 1).

### Calculation of the Model-Based Infection Ratios

**FIGURE 2.**
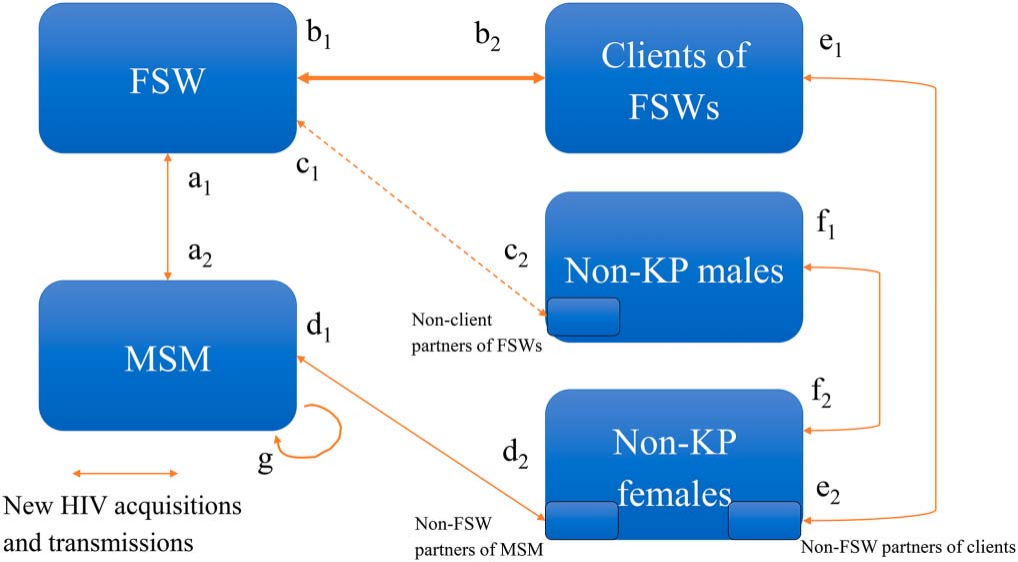
Simplified diagram of the number of new HIV acquisitions/transmissions occurring over a year between commonly modeled risk groups (large blue boxes). Several models (n = 7) represented intermediate-risk non-KP populations (eg, men reporting many stable or casual partners but not having paid for sex), but none of the models explicitly represented non-KP partners of KPs (small light blue boxes). Therefore, the ratios of new HIV infections among clients and partners of FSWs over the ones of FSWs in 2020 were first calculated using approach A, which considers the number of acquisitions by FSW clients in the numerator $\frac{b_{2}+e_{1}}{a_{1}+b_{1}+c_{1}}$, and/or the approach T based on direct transmissions in some models (eg, ratio of $\frac{b_{2}+c_{1}}{a_{1}+b_{1}+c_{1}}$ for clients and non-KP partners of FSW), or on counterfactual scenarios without transmission from a specific group to their non-KP partners over 2020 ($b_{2}=0;c_{2}=0$) for clients and partners of FSW, $d_{2}=0$ for cisgender female partners of MSM, or $e_{2}=0$ for non-KP partners of FSW clients, which subsequently reduces the number of secondary transmissions over that year. The approach A could only be used for clients and non-KP partners of FSW, which most models distinguish as a default group, whereas the models do not explicitly distinguish non-KP partners of other KP (represented here by small blue boxes). The line between *c*_1_ and *c*_2_ is dashed because a third of models assume no partnerships between FSWs and non-KP males.

The 2022 UNAIDS Global AIDS Update presents estimates of the “distribution of new HIV acquisitions” by risk group, including MSM, SW (which are almost exclusively informed by data on FSW), PWID, and “clients and partners of each KP”. In theory, the latter risk group could include all NIs acquired by any non-KP partners of each KP (eg, for clients of FSW: infections acquired during sex with FSW and noncommercial casual partners). However, the 2022 UNAIDS approach used to calculate the number of NIs occurring among clients and partners of KP over a year only reflects the total number of NIs transmitted by KPs to their non-KP partners (ie, excluding infections acquired from non-KP partners). Therefore, our model-based estimates for the *infection ratios* were calculated from the available model outputs using 2 approaches: either (1) dividing the number of NIs *acquired by non-KP* partners from any of their KP or non-KP partners by the number of NIs in the referent KP (approach A) and/or (2) dividing the number of NIs *transmitted by a KP* to their non-KP partners by the number of NIs in the referent KP (approach T, similar to the methodology used by the UNAIDS up to 2022 to derive the ratio, Fig. 2). Transmissions between 2 KPs were never considered when calculating *infection ratios*. The difference in the 2 approaches is the inclusion of FSW client's acquisitions from other non-KP partners (in addition to those from FSW) in approach A, whereas the approach T includes FSW transmissions to both nonclient partners and clients (but does not consider FSW clients' acquisitions from other non-KP partners). Where possible (for n = 127 settings), both approaches were used to calculate the ratio for the same model/setting/KP combination, but the approach A could only be used for clients and non-KP partners of FSW (Fig. 2). Where possible, estimates of the number of transmissions from a KP to their non-KP partners were preferentially calculated using the annual number of direct transmissions from the KP to their partners. Otherwise, estimates were derived by comparing the model-predicted cumulative number of NIs in 2020 with the corresponding number in a counterfactual scenario blocking all transmissions from the KP to their non-KP partners over the year (Table 1 and Fig. 2).

Table 1 reports how many estimates were available for each KP and which approach (*A* vs *T*) was used to derive these estimates for each model. Table 2, Supplemental Digital Content, http://links.lww.com/QAI/C158 reports the number of estimates by region and model. Because information on HIV acquisitions among nonclient partners of FSW was only available for the *Goals 2022* model, the *infection ratio* for clients and partners of FSW using approach A was always calculated by dividing the number of NIs acquired by clients of FSW by the number of NIs among FSW over the same year.

### Analysis of Infection Ratios

We derived the median and interquartile range (IQR) across available model-based estimates of the *infection ratios* for 2020 for each region/KP combination to summarize typical and country heterogeneities in ratios and compared these ranges to the UNAIDS Global AIDS Update 2022 ratio assumptions.^4^

Because temporal trends in model estimated infection ratios could reflect changes in epidemic dynamics and contributions of KPs to NIs, we compared 2020 and 2010 ratio estimates when available (10 models, Table 1). Correlation between 2010 and 2020 *infection ratios* estimates were assessed using Pearson correlation tests, whereas changes over time were assessed using bootstrapped paired *t* tests.

The influence of using different models or methods on ratio estimates was evaluated by (1) comparing *infection ratio* estimates by KP/region/model combination, combining all estimates from the 3 Goals models (*Goals 2022*, *Goals GF*, and *Goals HIV Prevention Trials Network*), and the non-Goals/AEM/Optima models into an *Other models* category (as most of these were for South Africa), (2) evaluating how *infection ratios* for clients and partners of FSW in South Africa depend on the modeled size of the FSW client population (which differed across models), and (3) comparing *infection ratios* using number of acquisitions by clients of FSW (approach A) with ratios using number of transmissions from FSW (approach T) in cases where both were available. Additionally, a sensitivity analysis (detailed in the supplement) compared numbers of direct HIV transmissions between a referent KP and their non-KP partners over a year, with estimates made through comparing to a counterfactual model scenario for the same model and year.

## RESULTS

### Comparison of Model KP Infection Ratios With the UNAIDS Assumptions From 2022

The median of all model-based estimates for the *infection ratios* for clients and partners of FSW across all regions was 0.7 (IQR: 0.5–1.0; n = 172 settings) when based on all HIV acquisitions by clients of FSW only (approach A) and 1.2 (0.8–1.8; n = 127) when based on transmissions from FSW to their clients and other non-KP partners (approach T, Table 2). The model-based estimates in Asia and Pacific (AP) using approach A (0.8; 0.5–1.6; n = 33) and using approach T (1.4; 1.1–2.1; n = 32) were much higher than the 2022 UNAIDS assumption (0.25). Similarly, model-based estimates in the Eastern Europe and Central Asia and Middle East and North Africa regions were approximately 3-fold higher than the previous UNAIDS assumptions. Model-estimated ratios for non-KP partners of MSM were 0.4 (0.2–0.6; n = 118). The model-based MSM ratios were lower than those previously assumed by the UNAIDS in ESA (model-based: 0.5; 0.2–0.8; n = 20; vs UNAIDS: 0.9). The median of model estimates for non-KP partners of PWID was 0.3 (0.2–0.6; n = 81), which was half that previously assumed by the UNAIDS for most regions (eg, 0.3; 0.2–0.4; n = 10 in ESA; vs UNAIDS: 0.8). Model-based ratio estimates for non-KP partners of TGW were much higher than the previous UNAIDS assumptions (model-based: 5.1; 1.2–7.0; n = 8; vs UNAIDS: 0.1 in all but 1 region). Finally, model-based estimates for non-KP partners of FSW clients were 1.1 (0.6–1.9; n = 112) overall and highest in WCA (1.7; 1.0–2.3; n = 29).

Model-based estimates of the *infection ratios* generally varied much less across regions than the UNAIDS 2022 assumptions (eg, <20% relative difference between median model estimates for clients and partners of FSW across regions, vs UNAIDS range of 0.25–1.5, Table 2). Median estimates of the ratios for female partners of MSM were similar in ESA and WCA (0.5), whereas the UNAIDS assumptions were higher for ESA compared with WCA (0.9 vs 0.5).

### Variation in Infection Ratios Over Time

**FIGURE 3.**
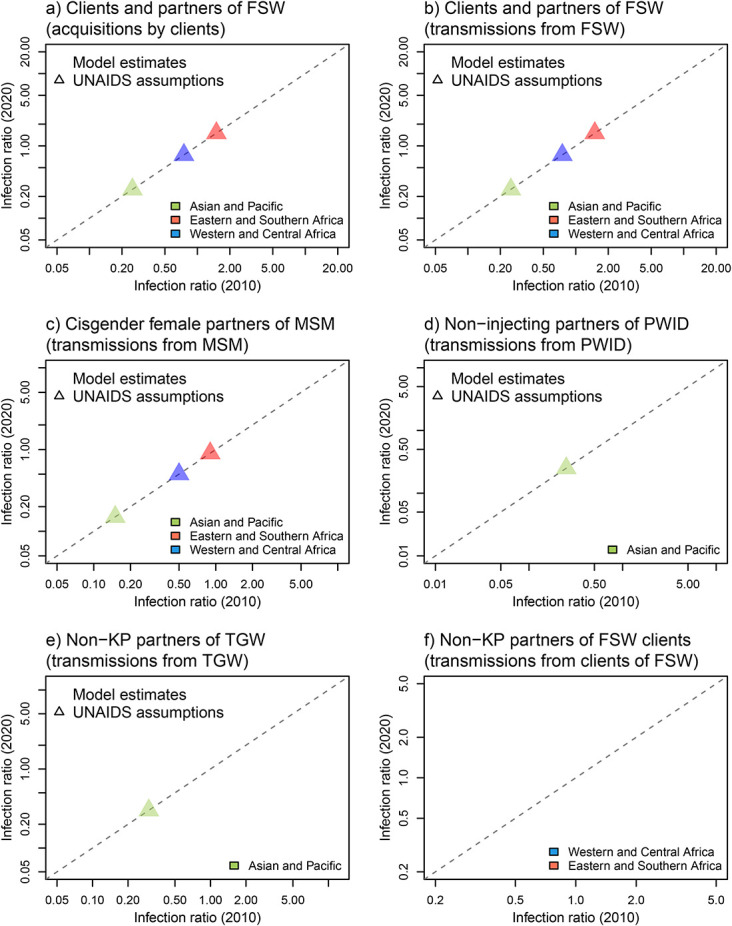
Comparison of *infection ratio* estimates over the years 2010 and 2020 for (A) clients and non-KP partners of FSW (using the number of infections acquired by FSW clients), (B) clients and non-KP partners of FSW (using the number of infections transmitted by FSW), (C) cisgender female partners of MSM, and non-KP partners of (D) PWID, (E) TGW, (F) clients of FSW, in the Asia and Pacific region (green), Western and Central African region (blue), and Eastern and Southern African region (red). Colored dots represent model estimates, whereas triangles correspond to 2022 UNAIDS assumptions for 2021 (assumed similar to 2010 because they do not’ include a time dimension). Estimates shown in the figures are those that were available for both 2010 and 2020, thus estimates from *Goals 2022* and *Goals GF* (only available for 2020) are not shown. The dashed diagonal line indicates perfect agreement between 2010 and 2020 estimates.

Although being strongly correlated, model-based *infection ratios* estimates often changed from 2010 to 2020 (Fig. 3, see Tables 3 and 4, Supplemental Digital Content, http://links.lww.com/QAI/C158). The estimated ratios for clients and partners of FSW decreased in almost all settings (by 20% on average), especially in ESA, with the largest relative decrease predicted by the *Thembisa* model for South Africa (from 22.6 in 2010 to 11.9 in 2020 for NIs acquired by FSW clients) partly because of the increasing proportion of NIs acquired by FSW over the period (Fig. 3A). Estimated ratios for female partners of MSM (available over time for 3 regions) and PWID were generally stable across the 2010 and 2020 time points, whereas those for TGW (only available over time for the AP regions) often increased over time (by 33% on average). Finally, around half of model estimated ratios for non-KP partners of FSW clients in ESA slightly increased over time (Fig. 3F) and the other half remaining constant or slightly decreasing over this period (eg, in WCA).

### Differences Between Estimated Ratios due to Underlying Models or Calculation Method

The *Goals* and *Optima* models generally produced much lower (∼5-times) median *infection ratios* estimates for clients and partners of FSW in ESA and WCA than other models (see Table 3, Supplemental Digital Content, http://links.lww.com/QAI/C158). However, all other models for ESA represented South Africa, where overall HIV prevalence is extremely high, which could have explained some of these model-related differences, although the *Goals/Optima* ratios for South Africa were similarly low compared with *Goals/Optima* estimates for the other countries of the region. The larger ratio estimates for the other models for South Africa were mainly due to the larger size of the FSW client population assumed in those models. One exception was the model by Mishra et al,^17^ where client population size was large (32%) but the estimated infection ratio was closer to the average (2.6) because large fractions of all NIs were acquired by FSW (11% vs 2%–3% in the other models, Figure 1, Supplemental Digital Content, http://links.lww.com/QAI/C158). The estimated ratios did not vary between the different *Goals* versions.

**FIGURE 4.**
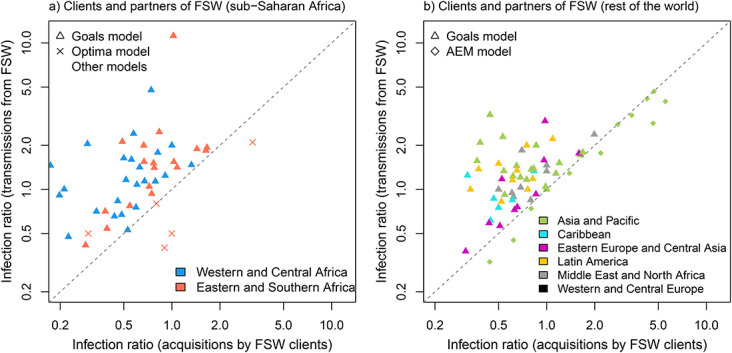
Comparison of 2020 *infection ratio* estimates for clients and partners of FSW based on model estimated new HIV infections among clients of FSW (approach A, *x* axis) and numbers of transmissions by FSW (approach T, *y* axis) from the same model/country combination in (A) sub-Saharan Africa, and (B) outside sub-Saharan Africa, stratified by model (geometric shapes) and regions (color). Dashed diagonal lines indicates perfect equality between estimated number of acquisitions among FSW clients and transmissions from FSW.

Model-based estimates of *infection ratios* for clients and non-KP partners of FSW differed between the 2 estimation approaches, with estimates based on transmissions from FSW to their partners (approach T) typically larger (by ∼1.7-fold) than those based on acquisitions among clients of FSW (approach A) (Table 2, Fig. 4). The largest difference was with *Goals 2022* for Mozambique where the number of NIs transmitted to non-KP by FSW over 2022 was 11-fold greater than the ones acquired by just clients over the same year. However, many models other than *Goals* estimated the opposite, with the largest differences (3- to –4-fold) reported by the *Thembisa*^15^ and Stone^10^ models for South Africa. This would suggest that most new HIV acquisitions among clients of FSW in South Africa occur during sex with non-FSW (which can be captured by approach A), due to the much larger prevalence of HIV among non-KP in the country compared with other global regions, and lower levels of condom use during non-commercial sex compared with commercial sex.

Sensitivity analysis suggests that using counterfactual scenarios instead of the direct number of transmissions by MSM and by FSW clients to their non-KP female partners only slightly overestimated the model-based UNAIDS infection ratios (by 1.1-fold on average, see Figure 2, Supplemental Digital Content, http://links.lww.com/QAI/C158).

## DISCUSSION

This analysis of 12 dynamic HIV transmission models of 106 countries located in 8 global regions found high ratios of new infections among non-KP partners relative to KP, across KPs, regions, for both 2010 and 2020–confirmed the importance of addressing KP treatment needs to reduce overall HIV incidence. We estimated substantive numbers of transmissions from clients of FSW to their non-FSW female partners, although there was heterogeneity in *infection ratios* across models. There were some qualitative agreements between the ratios used for the UNAIDS Global AIDS Update 2022 for clients and partners of FSW and model-based estimates. However, the UNAIDS assumptions for non-KP partners of MSM in ESA and (especially) PWID were higher than dynamic model estimates, which may have led to historic overestimation of fractions of all Nis that occurred among non-KP partners of KPs. There were substantial systematic differences across models particularly for estimates of the number of transmissions from FSW to their non-KP partners or acquired by FSW clients. Models indicated a large share of overall adult new HIV transmissions were from clients of FSW to partners who are not FSW, which were not considered among the “partners of KPs” in the UNAIDS reports yet have long been identified as an important population at risk when considering the impacts of HIV prevention for FSW and their clients, particularly for WCA.^9,21^

Differences between the 2022 UNAIDS assumptions and dynamic model-based estimates of the *infection ratios* varied across KP and regions. These differences initially seemed related to very heterogeneous assumptions by the UNAIDS for each specific KP compared with the median model-based estimates that were often similar across regions. However, median estimates hid substantial heterogeneities in model-estimated ratios between specific KP/region combinations, which may reflect actual differences in the dynamics of transmission between countries and differences between models. Clients and partners of FSW were the only population for which the medians of model estimates of *infection ratios* were qualitatively comparable with the UNAIDS assumptions in most regions, although *Goals* and *Optima* predicted much smaller (5-fold) ratios than the other models for SSA. The difference was striking for South Africa where large ratios (>5) were predicted by several models, including *Thembisa* that uses a broad definition of clients of FSW, which includes men who were previously clients (the other models do not), and assumes high numbers of partnerships between FSW clients and women at high risk (but who are not FSW). The largest *infection ratios* for clients and non-KP partners of FSW in the non-*Goals* and non-*Optima* models may be because these other models assumed that people at high risk of infection can also have short-term/casual noncommercial sexual partnerships, whereas the current *Goals* model assumes that KPs and FSW clients only form stable partnerships (and no short-term/casual partnerships) with non-KPs, resulting in lower *infection ratios*. Overall, time trends by KP were consistent with declines in HIV incidence, which have generally been greater in the male population than in the female population, largely because of greater female uptake of testing and treatment,^22^ thus averting transmissions to their male sexual partners.

Our results suggest that the UNAIDS *infection ratios* in the Global AIDS Update 2022 may have overestimated the proportions of NIs occurring among non-KP partners of MSM in ESA, and especially PWID globally, because their assumptions were often higher than model-based estimates. The latter should, however, be interpreted with caution (and not considered as “gold standard”) because (1) country-specific data characterizing the number of condom-protected/condomless sex acts between these KP and their non-KP partners is generally sparse, (2) estimates for partners of TGW were only available for 8 countries in the AP region, and (3) *AEM* estimated that over half of transmissions from TGW occurred during sex work (transmissions that could have been attributed to sex work).

Because *Goals* is applied to many countries for which there is scarce KP data, it is understandable that it uses conservative assumptions and fitting data that may not fully reflect existing country- or region-specific heterogeneities in levels of HIV acquisition risk and interventions. As an example, the current *Goals* model assumes a unique coverage of HIV viral load suppression (VLS) across all populations living with HIV, thus does not reflect wider gaps in HIV treatment often observed among specific KPs compared with non-KP.^23,24^ This can be important here because at high coverages levels of effective intervention (eg, VLS), small variation in coverages may translate into large differences in transmission risk to their partners. For example, VLS coverages of 90% in group A vs 95% in group B translate into twice higher “per capita” risk of HIV transmission by people in group A compared with group B. As a result, accuracy and robustness of future estimates of NIs acquired by non-KP partners of KP will improve when reflecting heterogeneity in sexual behaviors and coverage of HIV intervention by risk group, informed by reviews of regional data if no country-specific information is available. Clients of FSW are not classified as KP by the UNAIDS because they do not experience the same levels of vulnerability and stigma as other KPs,^25^ although it was estimated that they acquired and transmitted substantial number of Nis according to the models. Furthermore, differences between estimates of the number of acquisitions by FSW clients and the number of transmissions from FSW to clients in our analysis highlights the value of distinguishing infection estimates for clients of FSW from “other non-KP partners of KPs” and analyzing how relative proportions of infections among these groups vary over time and across countries.

This study has several important limitations. All estimates rely on mathematical models that are imperfect simplifications of population-level dynamics of infection transmission. Although modelers consider uncertainty in parameterization during their calibration process, this is not perfectly represented in our analysis, which only used model point estimates. In particular, model-based estimates of the *infection ratios* relied on estimates of the KP population sizes and fractions of all infections acquired by these KPs, for which we found important differences across models even for the same country (eg, Figure 1, Supplemental Digital Content, http://links.lww.com/QAI/C158 and Booton et al^26^). Many countries have scarce or no data about KPs, notably population sizes, coverage of ART among those living with HIV, VLS results among those on ART, and sometimes their historic prevalence trends, leading to uncertainties in model prediction and evaluation of their KP epidemic and response. No model-based estimate for the North American region was available, and only 1 estimate was available for the Western and Central Europe region; however, these regions account for less than 5% of the total number of Nis globally, indicating that this gap may have a limited impact overall.^22^ Very few estimates were available for non-KP partners of TGW, who themselves constitute a small fraction (2%) of the total number of Nis globally^4^ (although this may be underestimated because of missing surveillance data and absence of HIV programs for TGW in many countries). Moreover, KPs may report seasons of risk depending on occupation including sex work or dependency including injecting drugs.^27,28^ These dynamic risks often represent complexities that cannot be fully captured by surveys and mathematical models. Finally, counterfactual-based estimates for the number of transmissions by a KP to their non-KP partners, on which many model-based estimated ratios for female partners of MSM rely, are likely to be overestimated (because of accounting for onward transmission), but only slightly according to our sensitivity analysis.

Our analysis builds on extensive modelling covering most countries globally and many KPs, and detailed epidemic models relying on exhaustive reviews of country-specific empirical demographic and epidemiological data among KPs and non-KP partners. Using estimates from transmission-dynamic models allowed calculation of *infection ratios* over time, and these ratios for clients and other non-KP partners of FSW seemed to decrease over 2010–2020, likely due to interventions such as antiretroviral therapy (ART) having been more successful in reducing transmissions from FSW (ratio numerator) than acquisitions among them (ratio denominator). Our analysis provided insights into the epidemiological consequences of unmet HIV treatment needs of KPs (which are part of their own right to health) because they translate into ongoing HIV transmissions to non-KP (and other KPs), whose magnitude is usually poorly quantified.

In conclusion, our analysis highlighted weaknesses that led to improvements in the methodology used by the UNAIDS to calculate the distribution of acquisition of new HIV infection by population for its 2023 round of estimates.^5^ This included using existing models to directly estimate the fractions of all new HIV acquisitions acquired by FSW clients (without using *infection ratios*). Large differences across ratios from different models emphasize the need to promote additional epidemic model comparison exercises, which could improve our understanding of the influence of model assumptions and parameters on epidemic metrics, which are increasingly used to guide countries and agencies in their responses to HIV.
